# Liver X receptors agonist GW3965 re-sensitizes gefitinib-resistant human non-small cell lung cancer cell to gefitinib treatment by inhibiting NF-κB *in vitro*

**DOI:** 10.18632/oncotarget.15007

**Published:** 2017-02-02

**Authors:** Yong Hu, Jialan Zang, Haixia Cao, Ying Wu, Dali Yan, Xiaobing Qin, Leilei Zhou, Fan Fan, Jie Ni, Xiaoyue Xu, Huanhuan Sha, Siwen Liu, Shaorong Yu, Zhuo Wang, Rong Ma, Jianzhong Wu, Jifeng Feng

**Affiliations:** ^1^ Department of Clinical Cancer Research Center, Jiangsu Cancer Hospital, Jiangsu Institute of Cancer Research, Nanjing Medical University Affiliated Cancer Hospital, Nanjing, Jiangsu Province, China; ^2^ Department of Oncology, The First Hospital of Harbin City, Harbin, Heilongjiang Province, China

**Keywords:** non-small cell lung cancer, liver X receptor, GW3965, gefitinib resistance, NF-κB

## Abstract

The recent research shows that the inhibition of the nuclear factor-κB (NF-κB) pathway is a promising therapeutic option for patients who progress after treatment with the novel mutant-selective EGFR-TKIs. For propose to find a nontoxic drug to reverse the acquired gefitinib resistance, we examined whether the Liver X Receptors agonist GW3965 affect gefitinib resistance of HCC827/GR-8-2 cells. Cell viability was measured by CCK-8 assay. Levels of NF-κB, p-AKT and caspases were detected by Western blot analysis. Immunocytochemical analysis was used to detect the expression of NF-κB, p-AKT intracellularly. Induction of apoptosis and cell cycle arrest was measured by Flow cytometry assay. And results revealed that more than 90% of HCC827/GR-8-2 cells lived upon treatment with gefitinib at a dose of 5μM for 48h. However, when under the combine treatment of GW3965 (5μM) & gefitinib(5μM), cell death rate was increased observably. Co-administration of gefitinib & GW3965 induced cell apoptosis and cell cycle arrest. Additionally, we observed a dose-dependent- down-regulation of NF-κB in HCC827/GR-8-2 cells treated with gefitinib & GW3965. GW3965 and gefitinib synergistically decreased cell proliferation and induced apoptosis by inhibiting NF-κB signaling pathway in gefitinib resistant cells. These findings support our hypothesis that GW3965 could act as a useful drug to reverse the gefitinib resistance.

## INTRODUCTION

### Non-small cell lung cancer and gefitinib resistance

Lung cancer now is the leading cause in cancer-related mortality worldwide [1]. Among the several different pathologic types, non-small cell lung cancer (NSCLC) has been the most common lung cancer type [2]. About 10% of NSCLC patients can be identified the mutation in the tyrosine kinase domain of the epidermal growth factor receptor (EGFR) gene. Those patients have a rapid and generally clinical response to EGFR tyrosine kinase inhibitors (TKIs) such as gefitinib [3]. Gefitinib, as the first generation EGFR tyrosine kinase inhibitors (EGFR-TKIs), has been developed to be the recommended treatment to those NSCLC patients which harbor activating EGFR mutation [4]. The clinical use of EGFR-TKIs for advanced-stage NSCLC patients has revolutionized the treatment of the disease (disease control can be achieved in about 80% of the patient) [5]. Nonetheless, most patients who initially sensitive to gefitinib will develop acquire resistance within 6-12 months of therapy [6]. Drug resistance is a major obstacle in the NSCLC therapy. The EGFR mutations, PTEN loss, PI3K point mutations and other genetic lesions providing core pathway in NSCLC [7]. Until today, there is no effective strategy that has been found to overcome gefitinib resistance.

### LXR

LXRs are nuclear receptors with mounting evidence pointing to functional roles in a variety of malignancies recently. And LXRs are members of the nuclear receptor family. LXRα (also known as NR1H3) is expressed in metabolically active tissue. And LXRβ (also known as NR1H2) is expressed ubiquitously [8]. Targeting LXRs is a promising strategy for cancer therapy. LXRs agonist will effect on the tumor environment and the receptor status [9]. An earlier study demonstrated that the synergy between the activated LXR and EGFR antagonists act as a critical target regulating the growth of cancer cells [10]. And previous research had shown that the LXR agonist GW3965 could inhibit the NF-κB transcriptional activity [11]. Based on the close relationship of LXR and downstream of EGFR signaling pathway, and furtherly considering our preliminary results, we hypothesis that LXR agonist GW3965 can reverse the acquired gefitinib resistance by inhibiting NF-κB expression, and the research would provide a useful option for clinical treatment of acquired gefitinib resistant lung cancer.

## RESULTS

### HCC827/GR-8-2 cell line remains gefitinib resistance but did not have T790M mutation

**Figure 1:**
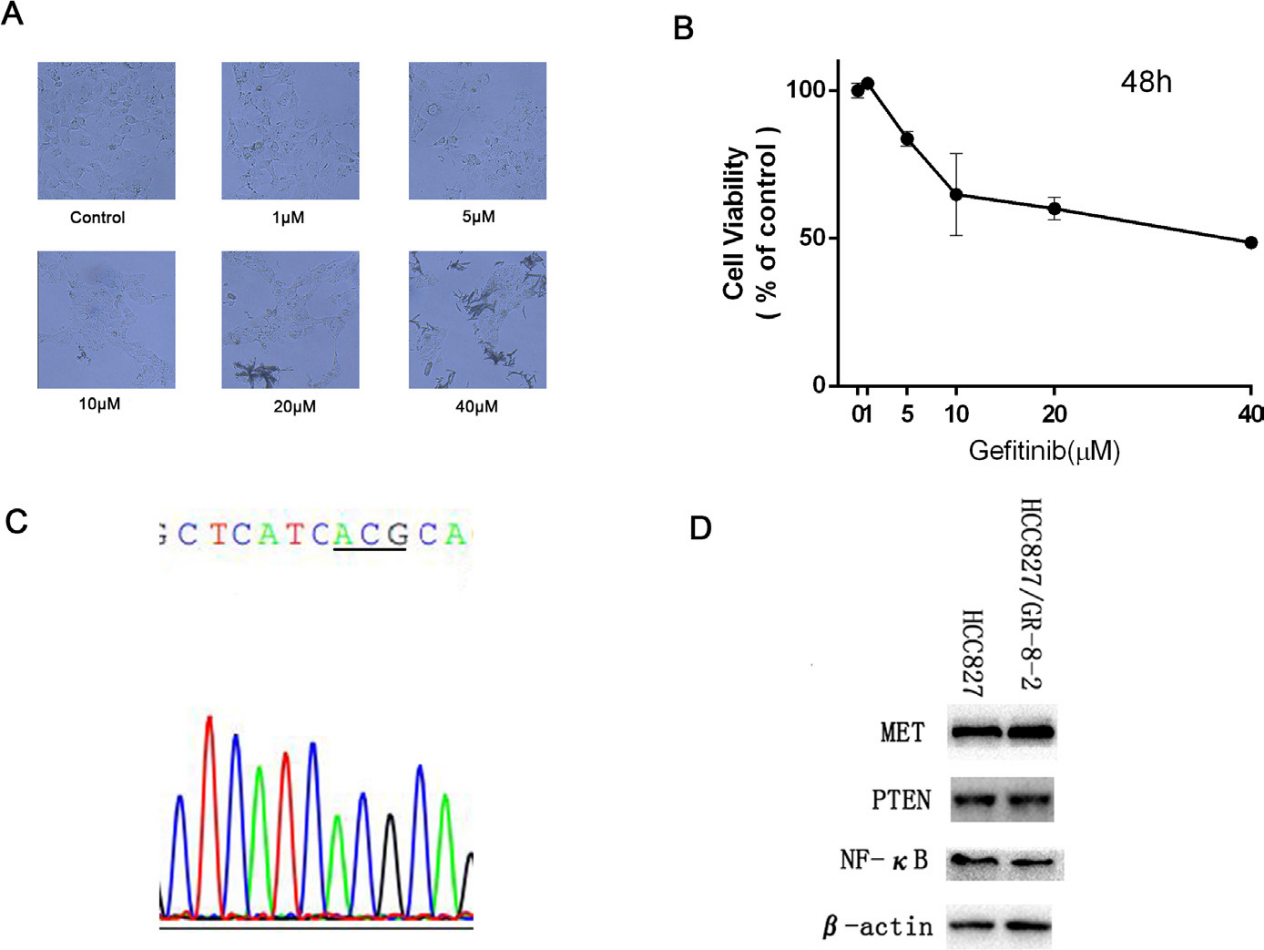
HCC 827/GR-8-2 cells resistance to gefitinib **A**. HCC827/GR cell were treated with gefitinib (1μM, 5μM, 10μM, 20μM, 40μM) for 48 hours. The results show that the HCC827/GR-8-2 cells were resistance to gefitinb. And in the high drug concentration (due to the high concentration, we can find the crystal of gefitinib), the cells turned round. **B**. The IC_50_ value of HCC827/GR-8-2 was 34.324±1.36 μM. **C**. We did not find the common T790M mutation in HCC827/GR-8-2 cells with the using of direct sequencing. **D**. The expression of Met, PTEN and NF-κB between HCC827 cells and HCC827/GR-8-2 cells were detected by western blot analysis. No obvious difference was observed.

We already established the EGFR-TKI-resistant cell lines [12]. While HCC827 cell line was still sensitive to gefitinib, HCC827/GR-8-2 was highly resistance to gefitinib (Figure 1A). From the results, the gefitinib IC_50_ values of HCC827 cell line and the HCC827/GR-8-2 cell line were (72.32±1.98)×10^-3^ μM and 34.324±1.36 μM respectively (Figure 1B). Gefitinib significantly suppressed cell growth in HCC827 cell line. Compare with the parental HCC827 cell line, the HCC827/GR-8-2 cell line were 474.6 folds more resistance to gefitinib.

We conducted direct DNA sequencing of EGFR at exons 18-22 on the HCC827/GR-8-2 cell line to examine the common mechanisms of acquired gefitinib resistance, the T790M mutation. But the T790M mutation was not observed in our HCC827/GR-8-2 cell line (Figure 1C). The EGFR expression was not significantly different between HCC827 cells and HCC827/GR-8-2 cells. And the expression of EGFR in HCC827/GR-8-2 cell line was almost the same (Figure 1D). We found that NF-κB expression was not significantly different between HCC827 cells and HCC827/GR-8-2 cells. Also did the MET, there was not obviously different between HCC827 cells and HCC827/GR-8-2 cells (Figure 1D).

### Expression of liver X Receptor in GW3965 treated HCC827/GR-8-2 cells

**Figure 2:**
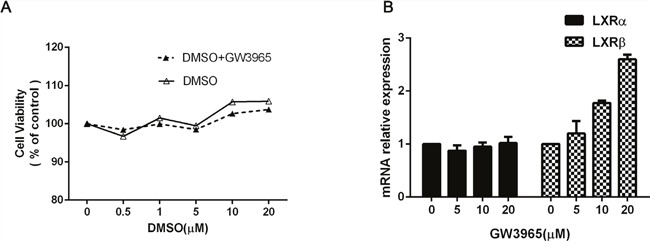
The effect of GW3965 **A**. HCC827/GR-8-2 cells were treated with GW3965 and/or DMSO for 48 hours (0.5μM, 1μM, 5μM, 10μM, 20μM). And the GW3965 cannot inhibit the cell proliferation. **B**. HCC827/GR-8-2 cells were treated with GW3965 for 48h. The total RNA was harvested from these cells to examine the LXRα mRNA and LXRβ mRNA expression. GW3965 could activate LXRβ significantly, while the LXRα mRNA expression did not change a lot.

Next, we investigated whether GW3965 could inhibit the cell viability or not. Different concentrations of GW3965 (1, 5, 10, 20 and 40 μM) were added in the HCC827/GR-8-2 cells for 48h.The results show that GW3965 have little effect on the cell viability in HCC827/GR-8-2 cells (Figure 2A). To further investigate the mechanisms of GW3965′s effect and its modulation on the LXRα and LXRβ mRNA, RT-PCR were conducted. Previous study already revealed that GW3965 can up-regulate the expression level of LXRβ mRNA [12]. Similarly, we find that the up-regulation of the expression level of LXRβ mRNA in a time-dependent manner. As shown in (Figure 2B), GW3965 significantly increased the expression of LXRβ mRNA, while the LXRα mRNA expression did not change a lot.

### Effects of GW3965 and the combination of gefitinib with GW3965 on HCC827/GR-8-2 cell viability

**Figure 3:**
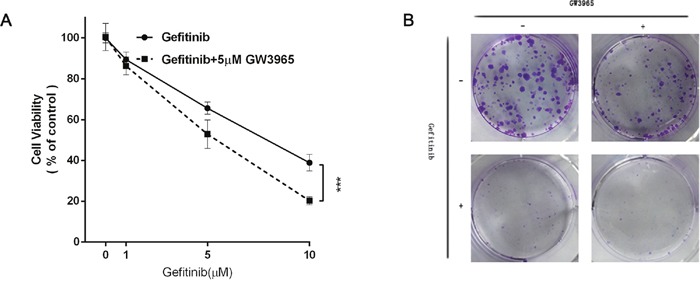
GW3965 reversing gefitinib resistance **A**. The cell viability was measured by cell counting kit 8 assay. GW3965 (5μM) replenishment re-sensitizes HCC827/GR-8-2 cells to Gefitinib (5μM) treatment (Two way ANOVA, p value< 0.0001). **B**. The colony formation assay of the HCC827/GR-8-2 cells: the cells were treated with the negative control, GW3965 (1μM), Gefitinib(1μM) and the combination treatment of GW3965(1μM) with Gefitinib(1μM) for two weeks. The cloning efficiency of the colony formation assay: the combination of GW3965 and gefitinib could markedly decrease the HCC827/GR-8-2 cells growth.

Herein, we co-conducted GW3965 (1, 5, 10μM) and gefitinib (5μM) on HCC827/GR-8-2 cells for further analysis whether GW3965 could overcome the gefitinib resistance or not. These CCK-8 assay results suggested GW3965 plus gefitinib significantly suppressed cell viability (Figure 3A). GW3965 had synergistic effects on reversing the gefitinib-resistance (p<0.0001). And the colony forming assays further proved that GW3965 (5μM) could decrease the drug-resistance cell proliferation. Same results were observed in gefitinib (5μM) treated group. But the combination of GW3965 (5μM) and gefitinib (5μM) could markedly (p<0.01) decrease the gefitinib-resistant cell growth (Figure 3B).

### GW3965 increases gefitinib-induced apoptosis and cell cycle arrest in HCC827/GR-8-2 cell line

**Figure 4:**
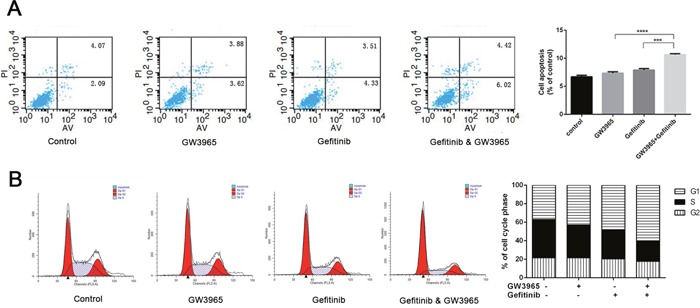
Flowcytometry revealed GW3965 induced apoptosis and G1/S cell cycle arrest **A**. When treated with 5 μM gefitinib or 5 μM GW3965, the cell apoptosis rate was not significantly different from control group. But cell apoptosis rate was higher in cells treated with gefitinib 5μM &GW3965 5μM compared with the single drug groups. And the p value <0.001(gefitinib 5μM verse the gefitinib 5μM &GW3965 5μM). **B**. The combine treatment of GW3965 and gefitinb induced the G1/S cell cycle arrest in HCC827/GR-8-2 cells.

We used flow cytometry to prove that GW3965 could increase cytotoxicity of gefitinib. As shown in (Figure 4A), when treated with 5 μM gefitinib, the cell apoptosis rate was not increased compared with the control group. And the apoptosis cells in GW3965 (5 μM) –treated group was not increased, either. The differences in the proliferation rates between the GW3965 (5 μM) group and the GW3965 (5 μM) with gefitinib (5 μM) group were significant (both P<0.001) (Figure 4A). The inhibition rate was increased (*P* < 0.001). The results demonstrated that GW3965 could significantly increase the apoptosis which induced by gefitinib in drug-resistant cells. And also, as shown in (Figure 4B), GW3965 could induce the increasing in the G1 phase population in HCC827/GR-8-2 cell line. S phase arrest along with a significant decrease in the number of cells was observed after treatment with the GW3965 (5 μM) and gefitinib(5 μM) for 48h. The percentages in the S phase were decreased. The results revealed that GW3965 could enhance cell cycle arrest when co-treated with gefitinib.

### GW3965 re-sensitizes gefitinib treatment by suppressing NF-κB expression in HCC827/GR-8-2 cell line *in vitro*

**Figure 5:**
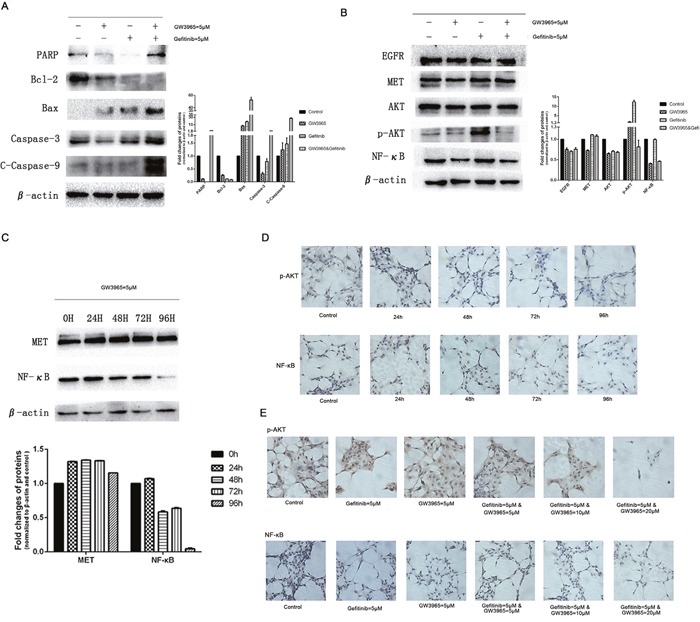
GW3965 sensitizes gefitinib by inhibiting NF-κB activation **A**. In combination of gefitinib, GW3965 enhances apoptosis of HCC827/GR-8-2 cells. The expression of caspase 3 and C-caspase 9 were detected as well as the Bcl-2, Bax and PARP. The IOD analysis show the fold changes of the PARP, Bcl-2, Bax, caspase 3 and C-caspase 9. **B**. GW3965 sensitizes gefitinib by AKT and NF-κB expression. When treated with gefitinib, AKT and NF-κB were activated. And GW3965 could inhibit the activation of them when the existence of gefitinib. And GW3965 could not influence the expression of EGFR and Met. **C**. The HCC827/GR-8-2 cells were treated with GW3965 (5μM) for 0h, 24h, 48h, 72h and 96h. The activation of NF-κB intracellular was inhibited significantly by GW3965. But the expression of Met was not change a lot. **D**. The immunohistochemistry were performed to detect the expression of NF-κB and p-AKT intracellular. **E**. The HCC827/GR-8-2 cells were treated with negative control, GW3965 (5μM), gefitinib (5μM), gefitinib (5μM) & GW3965 (5μM), gefitinib (5μM) & GW3965 (10μM), gefitinib (5μM) & GW3965 (20μM) for 72h. As the doses of GW3965 grew, the inhibition of the NF-κB and p-AKT expression became more significantly.

Evidence indicates that the NF-κB pathway plays an important role in the development of chemo-resistance [13]. Also, NF-κB plays an important role in the induction of apoptosis and drug resistance in cancer cells [14]. The inhibition of NF-κB can alter a number of downstream signals including the suppression of anti-apoptotic proteins and induction of apoptosis [15]. To determine whether GW3965 reversed gefitinib-resistance via apoptosis, we next explore the effect of GW3965 and/or gefitinib on NF-κB and caspases level in cells. We detected the apoptosis-related protein expression. Western blot analysis showed that PARP, bax, caspase-3 and C-caspase-9 was up-regulated significantly in GW3965 plus gefitinib-treated group compared with GW3965 or gefitinib-treated groups (Figure 5A). On the contrary, the anti-apoptotic proteins bcl-2 intracellularly was decreased markedly upon the combination treatment of GW3965 with gefitinib compared with GW3965 or gefitinib alone. NF-κB expression was decreased obviously among GW3965 plus gefitinib-treated groups compared to control or gefitinib-treated groups (Figure 5B). We treated HCC827/GR-8-2 cells with GW3965 (5μM). And NF-κB expression was markedly deceased in GW3965-treated cells in a time depending manner. And the drug treatment seemed have little effect on the MET expression (Figure 5C). The combination of GW3965 with gefitinib could significantly inhibit the NF-κB expression compared to the gefitinib-treated cells. Moreover, the immunocytochemical proved that GW3965 inhibited NF-κB expression in a time (Figure 5D) and dose depending manner (Figure 5E) in HCC827/GR-8-2 cell line. Recent research showed that the NF-κB could be activated following AKT phosphorylation [16]. Moreover, p-AKT expression was significantly up-regulated when cells were treated with gefitinib. As shown in Figure 5D, 5E, in combination with GW3965, the expression of p-AKT was down regulated compared with gefitinib-treated group. Above results indicated that GW3965 combined with gefitinib has a synersgistic effect. Our *in vitro* studies investigating that the NF-κB expression and consequent tumor cell survival can be suppressed by LXR ligands GW3965.

### The specific inhibition of NF-κB down-regulate the gefitinib resistance

**Figure 6:**
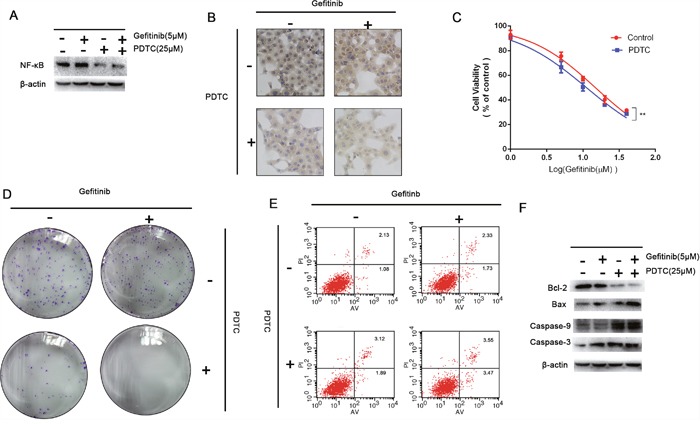
Inhibition on the expression of NF-κB **A**. The expression of NF-κB intracellularly could be inhibited significantly by PDTC. **B**. The immunohistochemistry were performed to detect the expression of NF-κB intracellularly. The HCC827/GR-8-2 cells were treated with PDTC (25μM). And the expression of NF-κB was significantly decreased which was dependent by dose. **C**. Cells treated with gefitinib (5μM), gefitinib (5μM) & PDTC (25μM), for 72h. The CCK-8 results showed that the inhibition of NF-κB can decrease the gefitinib resistance significantly. **D**. The cloning efficiency of the colony formation assay showed the PDTC-induced-inhibition of NF-κB could markedly decrease the cells growth when the presence of gefitinib (5μM). **E**. Flow cytometry revealed the inhibition of NF-κB induced apoptosis, especially when the presence of gefitinib (5μM) (P<0.05). **F**. The expression of caspase 3 and caspase-9 were detected as well as the Bcl-2 and Bax. The inhibition of NF-κB can increase the caspase-3 level.

PDTC can specific decrease intracellular expression level of NF-κB in dose dependent manner [17]. Indeed, the expression levels of NF-κB were investigated in PDTC-treated NSCLC cell lines. For this purpose HCC827/GR-8-2 cells were treated with different concentrations of gefitinib, and with combined treatment of PDTC (25 μM). Indeed, we observed that in our experimental conditions PDTC and gefitinib decreased the drug resistance significantly (Figure 6A). As shown in Figure 6B, in comparison to control, a significantly decrease in the expression level of NF-κB was observed in the cells after treatment with 25 μM PDTC for 72h. While the single agent gefitinib could not decrease the expression of NF-κB, the combination of PDTC (25 μM) with gefitinib significantly decreased the concentrations of intracellular NF-κB respectively (Figure 6B). We further aimed to determine the influence of PDTC on the gefitinib sensitivity by identification of IC50 values under the drug treatments. CCK-8 assay (Figure 6C) results showed the gefitinib IC_50_ values in the control group, and the PDTC (25 μM) group were 14.84 μM, 11.18 μM, respectively. The colony formation assay showed the inhibition of NF-κB can markedly attenuate the cell proliferation (Figure 6D). Flow cytometry analysis showed remarkable increase of early apoptotic cells upon the inhibition of NF-κB. Figure 6E show the treatment of PDTC induced the apoptosis. The inhibition rate was significantly higher in cells treated with PDTC group than in cells that were only treated with gefitnib. With the co-treating of PDTC and gefitinib, the percentage of apoptosis was increased remarkable (Figure 6E). While both agents decreased the cells viability in dose dependent manner, caspases changes clearly indicated synergy action of gefitinib and PDTC (Figure 6F). As treated with PDTC, the expression levels of caspases were increased compared with the control group. And the apoptotic proteins in PDTC–gefitinb-treated group were increased, too. The results suggested the inhibition of NF-κB had significantly effects on reversing the gefitinib-resistance.

### Rescue assay: The activation of NF-κB can attenuate the GW3965-induced re-sensitize of gefitinib

**Figure 7:**
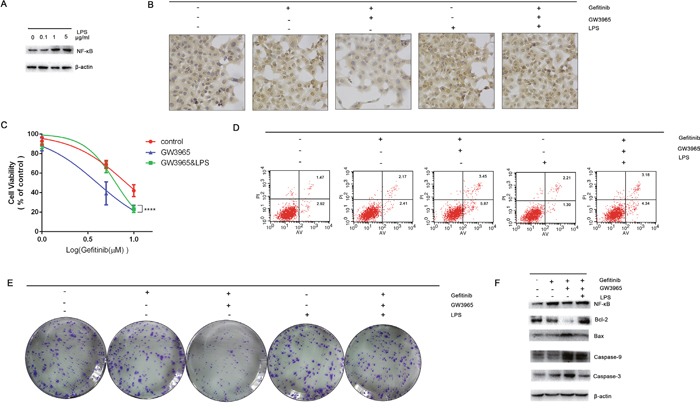
The activation of NF-κB **A**. LPS (2μg/ml) can significantly activate the expression of NF-κB intracellular. **B**. The immunohistochemistry were performed to detect the expression of NF-κB intracellular. LPS can significantly activation the expression of NF-κB. And LPS can reverse the GW3965-induced NF-κB inhibition. **C**. Cells treated with gefitinib (5μM), gefitinib (5μM) & GW3965 (5μM), gefitinib (5μM) & GW3965 (5μM) & LPS (2μg/ml) for 72h. The CCK-8 results show that the activation of NF-κB can increase the IC50 value of cells to gefitinib (p<0.05). **D**. But the cloning efficiency of the colony formation assay show the LPS-induced-activation of NF-κB could not increase the cells growth, so does when the presence of gefitinib (5μM). **E**. Flowcytometry revealed the LPS cannot decrease the cell apoptosis, but can decrease the gefitinib & GW3965 induced cell apoptosis (P<0.05). Activation of NF-κB can attenuate the GW3965-induced gefitinib resistance reversing. **F**. The expression of caspase 3 and caspase 9 were detected as well as the Bcl-2 and Bax. The activation of NF-κB can decrease the caspase-3 level.

LPS stimulation results in the up-regulation of NF-κB [18]. We next examined whether LPS stimulus is able to increase the protein levels of NF-κB. We further aimed to determine the influence of LPS on the GW3965-induced reversing of gefitinib resistance by identification the important role of NF-**κ**B in gefitinib resistance. For this purpose gefitinib resistance cells were treated with LPS(2μg/ml), gefitinib(5 μM), and with combined treatment of LPS & gefitinib in concentrations selected. A strong LPS induced activation of NF-**κ**B was observed in cells (Figure 7A). Data are summarized in Figure 7B clearly showing that NF-κB was markedly higher in LPS-treated (2μg/ml) groups as compared to control group. The IC_50_ values of gefitinib in the gefitinib(5 μM) group, the GW3965 (5 μM) with gefitinib(5 μM) group, and the LPS(2μg/ml) and GW3965 (5 μM) with gefitinib(5 μM) group were 8.26 μM, 3.99 μM, and 6.36 μM, respectively (Figure 7C). These CCK-8 assay results suggested cellular resistance to gefitinib was restored after the treatment of the LPS(2μg/ml). Interestingly, LPS treatment did not result in decreased cell apoptosis. After optimization of the LPS dose (2μg/ml) and time course (72 h), the GW3965 with gefitinib-induced cell apoptosis rate was decreased (Figure 7D). On the other hand, LPS alone or concomitantly to gefitinib activated the NF-**κ**B activity indicating that apoptosis was a result of NF-κB inhibition. The colony formation assay showed the restored expression of NF-κB could up-regulate cell proliferation. When combined treatment of gefitinib and LPS and GW3965 was applied in mentioned dose, the percentage of cell colonies were significantly decreased (Figure 7E). And, LPS induced increase expression of NF-**κ**B can further decrease caspases proteins. The NF-**κ**B overexpression clearly showing that the expression levels of caspase 3, caspase 9 and bax, were markedly lower in cells compared with the gefitinib and LPS and GW3965 group (Figure 7F).

## DISCUSSION

NSCLC represents in strong need of new, effective therapeutic approaches to solve the EGFR-TKIs resistance. Surprisingly, with the using of GW3965 could not only re-sensitize the treatments of EGFR-TKIs but also might overcome the acquired resistance as the dose rise [12]. Evidences have emerged that activation of LXR inhibits various types of cancer cells’ proliferation [19]. More importantly, recent researches have been proved the LXR agonist could significantly inhibit the activation of EGFR downstream pathways, which indicates that LXR agonist is a potential drug that could reverse the acquired resistance to gefitinib [20]. Our preliminary study also demonstrated that addition of LXR agonist could inhibit lung cancer cells (resistance to gefitinib) proliferation, induce cancer cells’ apoptosis and significantly increase cancer cells’ sensitivity to gefitinib by the inhibition of AKT phosphorylation [12]. The activation of the LXR in different types of cancer cells resulted in significant cell apoptosis [21]. The results emerged from these studies raise the possibility that the nuclear receptor LXR may be a potential drug target in multiple cancers especially in NSCLC.

LXR could be activated by the agonist GW3965 [22]. And LXR agonist could reverse the increase of NF-κB-p65 protein expression [23]. Recent research demonstrated that GW3965 can suppress the activation of NF-κB in a dose-dependent manner. The p65 subunit of NF-κB binding to the NF-κB consensus site could be greatly inhibited when pretreated with GW3965 [24]. NF-κB family can activate a series of gene transcription and control cell signaling pathways and affect cellular functions in a wide range of biological process [25]. As a transcription factor complex, NF-κB could be activated by cellular stresses, such as chemotherapeutic agents; upon induction, NF-κB translocate to the nucleus where it binds to specific DNA sequences in target genes involved in carcinogenesis, cell survival, and growth regulation [26]. NF-κB promote the tumor development and progression [27]. NF-κB activation will drive tumor cell survival [28]. Previous research also suggest that the NF-κB pathway correlates with the development of chemo-resistance, at least in part by the inhibition of NF-κB [29]. NSCLC containing EGFR mutation show the elevated NF-κB activity [30]. NF-κB activation could replace the oncogenic EGFR signaling pathway in the effective and persistent inhibition of target gene when EGFR harboring mutation [31]. Current studies reveal that NF-κB activation promoted resistance to EGFR-TKIs and might be a therapeutic strategy for EGFR-TKI resistance [32]. The NF-κB signaling pathway activation is a critical adaptive survival mechanism engaged by EGFR oncogene inhibitors [28]. Also, NF-κB is implicated in the induction of apoptosis in cancers, especially acquired drug resistance cancer cells [33]. NF-κB activation confers the generating of gefitinib resistance in lung cancer [34]. The inhibition of NF-κB is sufficient to reduce the viability of cells that acquired EGFR-TKI resistance. Also the inhibition of NF-κB can activate a number of downstream events including suppression of anti-apoptotic genes and induction of apoptosis [35].

**Figure 8:**
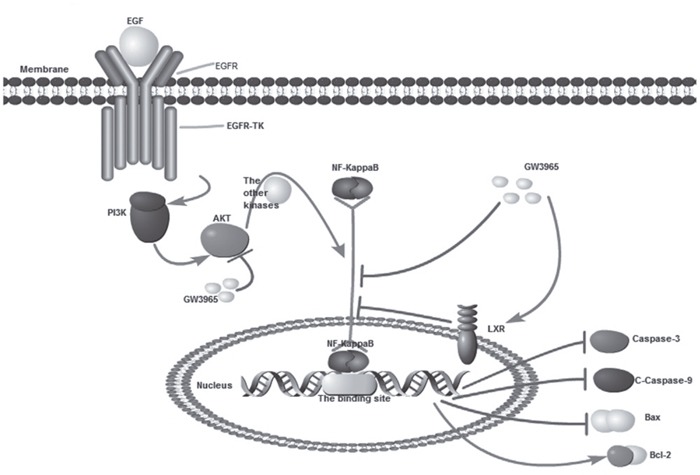
GW3965 inhibit NF-κB pathway (*By pathway builder software*).

The study we conducted showed the forced activation of the LXR with highly synthetic agonists GW3965 could inhibit the expression of NF-κB (p65) in a time dependent manner. NF-κB in HCC827/GR-8-2 cells was down-regulate markedly when treated with combination of GW3965 & gefitinib. Therefore, the down-regulation of NF-κB can also negatively regulate the expression of apoptosis proteins such as Bax, Caspase-3 and C-Caspase-9. The induction of apoptosis related proteins further regulated the resistant cells. This study demonstrates that NF-κB plays a significant role in acquired gefitinib resistance and the GW3965-induced inhibition of NF-κB will activate the downstream apoptosis gene products (Figure 8). For confirm the hypothesis that the GW3965-induced-reversing of resistance was the result of NF-κB inhibition, we furtherly conducted the NF-κB inhibition assays. Since the PDTC has been described to induce NF-κB inhibition and cell death in different types of cancer cells [36], intracellular NF-kB levels were evaluated after treatment with 25 μM of PDTC for 72 h. The expression level of NF-κB can be inhibited by PDTC, which is logically consistent with the previous findings [37]. However, our results revealed that the inhibition of NF-κB at the same time down-regulate the IC50 value of PDTC-treated cells to gefitinib. On the contrary, a strong LPS induced increased expression of NF-**κ**B was observed in gefitinib resistant cells. Interestingly, LPS treatment did not result in increased cell proliferation in HCC827/GR-8-2 cells. Nevertheless, treatment with LPS can restore the gefitinib resistance.

However, our findings do not entirely explain the mechanisms of that the liver X receptors agonist GW3965 re-sensitizes gefitinib-resistant NSCLC cell lines harboring EGFR mutant to gefitinib treatment. Future study will be needed to assess the efficacy and clinical utility of the LXR agonists such as GW3965 as a potential clinical candidate as they are becoming useful and available for clinical testing. The frequently adverse effects seen in TKI treatment require further investigation. A well-adjusted balance between efficacy and adverse effects is desirable. No effects on overall survival were reported. Therefore, further studies are required.

## MATERIALS AND METHODS

### Reagents and generation of gefitinib-resistance HCC827 cells

GW3965 and DMSO were purchased from Sigma-Aldrich (St. Louis, MO, USA). Cell counting kit-8 was purchased from Dojindo (Kumamoto, Japan). RPMI 1640 (Gibco) were supplemented with 10% fetal bovin serum (FBS) (Wisent, Canada). Gefitinib (Iressa, Gef) was purchased from AstraZeneca. Penicillin-Streptomycin Solution was purchased from Gibco. PDTC and LPS were purchased from Beyotime, Beijing. The antibodies against caspases, NF-κB, β-actin, AKT, p-AKT, PTEN, EGFR were purchased from Cell Signaling Technology (Danvers, MA, USA). HCC827 cell line is a non-small cell lung cancer cell line harboring the EGFR exon 19 deletion (Del E746-A750), and the cell line was purchased from Cell Bank, Shanghai Institutes for Biological Sciences, Chinese Academy of Sciences. And HCC827/GR cell lines were cultured in the Jiangsu Cancer Hospital. HCC827/GR cell line has been used as a gefitinib-resistance cell model. We exposed the HCC827 cells to increasing the concentration of gefitinib according to previously described method [38]. Finally, the HCC827 cells generated stable gefitinib resistance. HCC827/GR-8-2 cell line was isolated and it was confirmed resistance to gefitinib independently. The resistant cell lines were passed more than 25 times in the presence of gefitinib and maintained the resistance which was confirmed by CCK-8 assays. HCC827 cell lines were maintained concomitantly without gefitinib. So the parental cells HCC827 was still sensitive to gefitinib and we examined it every 5 passages. HCC827 cells and HCC827/GR cells were maintained in RPMI1640 supplemented with 10% heating-inactivated FBS and 1% Penicillin-Streptomycin Solution. Cells were maintained at 37°C in a humidified incubator with 5% CO_2_.

### Cell sensitivity assay

Cells were planted in 96-well flat bottom tissue culture plates at 5× 10^3^ cells/well and incubated for 24h. After 12h, the cells were treated with drugs according to the divided groups as following: 1) for gefitinib resistance test: gefitinib (1μM, 5μM, 10μM, 20μM, 40μM); 2) for the test of reversing of resistance: gefitinib (1μM, 5μM, 10μM) with GW3965 (5μM) or not; 3) for the PDTC treatment: gefitinib (1μM, 5μM, 10μM, 20μM, 40μM); gefitinib (1μM, 5μM, 10μM, 20μM, 40μM) & PDTC (25μM); 4) for the LPS treatment: gefitinib (1μM, 5μM, 10μM); gefitinib (1μM, 5μM, 10μM) & GW3965 (5μM); gefitinib (1μM, 5μM, 10μM) & GW3965 (5μM) & LPS (2μg/ml). After incubation, CCK-8 reagents were mixed with RPMI 1640 by a ratio of 1 to 10 and then added 110μl of the mix to each well. Incubated for 1-4h, then spectrometric absorbance at wavelength of 450nm was measured on using an ELISA plate reader. IC50 values were calculated according to the percentages. Each experiment was performed in triplicate and then averaged.

### Sequencing of the EGFR gene

The DNA was extracted from the HCC827/GR-8-2 cell lines using a QIA-amp DNA Mini Kit (Qiagen, Tokyo, Japan) to determine the EGFR sequence of the cell lines. The exons encoding the intracellular domain (exons 18-22) were amplified by PCR. Primer sequence were as follow (Table 1). The sequencing was conducted using an ABI 3500 sequencer. Three individual experiments were repeated.

**Table 1:** Sequence of EGFR at exons 18-22

Primer name	primer sequence 5’ to 3′
EGFR18-F	AGCATGGTGAGGGCTGAGGTGAC
EGFR18-R	ATATACAGCTTGCAAGGACTCTGG
EGFR19-F	CCAGATCACTGGGCAGCATGTGGCACC
EGFR19-R	AGCAGGGTCTAGAGCAGAGCAGCTGCC
EGFR20-F	GATCGCATTCATGCGTCTTCACC
EGFR20-R	TTGCTATCCCAGGAGCGCAGACC
EGFR21-F	TCAGAGCCTGGCATGAACATGACCCTG
EGFR21-R	GCTCCCTGGTGTCAGGAAAATGCTGG

### Colony formation assay

Cells were seeded at 400 cells per well in flat bottomed 6-well plates. After 24 hours of incubation, cells were treated with 2 ml culture medium with drugs as following groups: 1) for the test of reversing of resistance: gefitinib (1μM); GW3965 (1μM); gefitinib (1μM) & GW3965 (1μM) ; control group; 2) for the PDTC treatment: gefitinib (1μM); PDTC (1μM); gefitinib (1μM) & PDTC (1μM);3) for the LPS treatment: gefitinib (1μM); gefitinib (1μM) & GW3965 (1μM); LPS (2μg/ml); gefitinib (1μM)&GW3965 (1μM)&LPS (2μg/ml). After cultured for 2 weeks, cells were stained with hematoxylin. The experiments were performed for three times.

### Flow cytometry analysis for cell cycle and apoptosis analyses

For detecting apoptosis, the cells were seeded in flat bottomed 6-well plates. After 24h of incubation, cells were cultured in the medium of drug dilutions as following groups:1) for the test of reversing of resistance: control; gefitinib (5μM); GW3965 (5μM); gefitinib (5μM) & GW3965 (5μM); 2) for the PDTC treatment: control; gefitinib (5μM); PDTC (25μM); gefitinib (5μM) & PDTC (25μM);3) for the LPS treatment: control; gefitinib (5μM); gefitinib (5μM) & GW3965 (5μM/ml); LPS(2μg/ml); gefitinib (5μM/ml)&GW3965 (5μM/ml)& LPS(2μg/ml). Typsinized cells were washed with PBS. Apoptosis assay was performed to detect apoptotic cell with an Annexin V-FITC/PI double staining apoptosis detection kit with using the flow cytometer. Three individual experiments were repeated.

For detecting cell cycle, the cells were cultured in flat bottomed 6-well plates. After 24h of incubation, cells were cultured in the absence (control) or presence of gefitinib (5μM) and/or GW3965 (5μM). Typsinized cells were washed with iced-cold PBS and fixed in 75% ethanol at -20°C. Then, samples were incubated with 1U/ml of RNase A and 10μg/ml of PI for 15 min at room temperature in the dark and washed. And cell cycle analysis was accessed by flow cytometry (BD FACS Calibur, USA) acquiring 20,000 events that were analyzed by MODFIT3.0 software. Three individual experiments were repeated.

### Quantitative PCR

HCC827/GR-8-2 cells were treated with drugs for 48h. Then, total RNA was isolated by using Trizol reagent (Invitrogen) following the instructions. cDNA was synthesized with PrimeScript RT Master Mix(Takara, Dalian, China) according to manufacturer's instructions. The quantitative PCR was performed using SYBR Green PCR Mix (Roche, Mannheim, Germany). And β-actin was used as an internal control to normalize the amount of total RNA in each sample. The primer sequences of β-actin, LXRα and LXRβ were as Table 2. Relative levels of gene expression were determined using the ΔΔCt method. All reactions were repeated three times for each sample. Three individual experiments were repeated.

**Table 2:** Primer sequence of β-actin, LXRα and LXR

Primer name	primer sequence 5’ to 3′
β-actin-F	AGCACTGTGTTGGCGTACAG
β-actin-R	GGACTTCGAGCAAGAGATGG
LXRα-F	TCTGGAGACATCTCGGAGGTA
LXRα-R	GGCCCTGGAGAACTCGAAG
LXRβ-F	GTGGACTTCGCTAAGCAAGTG
LXRβ-R	ATGATCTCGATAGTGGATGCCT

### Immunocytochemical analysis

HCC827/GR-827-8-2 cells were seeded in 6-well chamber slides at 10×10^4^ per well. Then cells were treated with the drugs for 72h. And the divided treatment groups was as following: control; gefitinib (5μM); GW3965 (5μM); gefitinib (5μM) & GW3965 (5μM) ; 2) for the PDTC treatment: control; gefitinib (5μM); PDTC (25μM); gefitinib (5μM) & PDTC (25μM);3) for the LPS treatment: control; gefitinib (5μM); gefitinib (5μM) & GW3965 (5μM); LPS(2μg/ml); gefitinib (5μM) & GW3965 (5μM) & LPS (2μg/ml). The cells were fixed in 4% paraformaldehyde for 30minutes. Then the cells were treated with 20% hydrogen peroxide in methanol for 20min to inhibit the endogenous peroxidase activity. After blocked in 5% BSA in PBS for 1 hour, the cells were incubated with the primer antibodies(1:1000 dilution) NF-κB or p-AKT at 4°C for a night. After the washing of PBS, the cells were incubated with horseradish peroxidase-conjugated IgG antibody (cell signaling, USA) for a hour at temperature. Then the cells were incubated with the anti-rabbit biotin-conjugated secondary antibody for 1h after washing with PBS. Next, the cells were treated with Vectastain ABC reagent for 30 min at 4°C overnight, then the cells were stained with DAB and hematoxylin. Immunostained slides were scanned and selected regions of interest were outlined manually. Three individual experiments were repeated.

### Western blot analysis

Cells were lysed in RIPA buffer (Beyotime Biotechnology, China). Proteins were separated on 8% to 12% SDS-polyacrylamide gel electrophoresis gels and transfected to a polyvinylidene fluoride membrane. The PVDF membranes were blocked with 5% bovine serum albumin. Then the membranes were probed with primary antibodies and incubated in 4°C for a night. Primary antibodies used were NF-κB, p-AKT, AKT, PARP, Bcl-2, Bax, β-actin, EGFR, MET and caspases (1:1000, Cell signaling). After washing in PBS with 1% Tween-20, the membrane was incubated with secondary antibody (Cell Signaling, USA). The protein bands were visualized using the enhanced detector Chemiluminescence with BeyoECL Plus kits (Beyotime, China). Three individual experiments were repeated.

### Statistical analysis

The results of all experiments were expressed as mean ± standard deviation (SD) of at least three separate tests. Student's t-test or two-way ANOVA was performed with SPSS Version 21.0. P<0.05 was considered statistically significant and P<0.05 indicates a statistically significant difference. All data are presented as the mean and standard deviation from at least three separate experiments. P<0.05 was considered to indicate a statistically significant difference. All data are presented as the mean and standard deviation from at least three separate experiments. And a p value <0.05 was consider statistically significant. The statistical analysis software used was SPSS Version 21.0.
